# Coculture of *Trichoderma harzianum* and *Bacillus velezensis* Based on Metabolic Cross-Feeding Modulates Lipopeptide Production

**DOI:** 10.3390/microorganisms10051059

**Published:** 2022-05-20

**Authors:** Barbara Fifani, Sebastien Steels, Catherine Helmus, Alice Delacuvellerie, Barbara Deracinois, Vincent Phalip, Frank Delvigne, Philippe Jacques

**Affiliations:** 1 UMR Transfrontalière BioEcoAgro No 1158, University Lille, INRAE, University Liège, UPJV, YNCREA, University Artois, University Littoral Côte d’Opale, TERRA—Teaching and Research Centre, Gembloux Agro-Bio Tech, University Liège, 5030 Gembloux, Belgium; baro.fifani08@hotmail.com (B.F.); s.steels@uliege.be (S.S.); catherine.helmus@uliege.be (C.H.); f.delvigne@uliege.be (F.D.); 2 UMR Transfrontalière BioEcoAgro No 1158, University Lille, INRAE, University Liège, UPJV, YNCREA, University Artois, University Littoral Côte d’Opale, ICV—Charles Viollette Institute, 59000 Lille, France; barbara.deracinois@univ-lille.fr (B.D.); vincent.phalip@univ-lille.fr (V.P.); 3 Proteomics and Microbiology Department, University of Mons, 7000 Mons, Belgium; alice.delacuvellerie@umons.ac.be

**Keywords:** *Bacillus velezensis*, *Trichoderma harzianum*, microbial interaction, coculture, nutritional dependency, nitrogen, lipopeptides

## Abstract

Cocultures have been widely explored for their use in deciphering microbial interaction and its impact on the metabolisms of the interacting microorganisms. In this work, we investigate, in different liquid coculture conditions, the compatibility of two microorganisms with the potential for the biocontrol of plant diseases: the fungus *Trichoderma harzianum* IHEM5437 and the bacterium *Bacillus velezensis* GA1 (a strong antifungal lipopeptide producing strain). While the *Bacillus* overgrew the *Trichoderma* in a rich medium due to its antifungal lipopeptide production, a drastically different trend was observed in a medium in which a nitrogen nutritional dependency was imposed. Indeed, in this minimum medium containing nitrate as the sole nitrogen source, cooperation between the bacterium and the fungus was established. This is reflected by the growth of both species as well as the inhibition of the expression of *Bacillus* genes encoding lipopeptide synthetases. Interestingly, the growth of the bacterium in the minimum medium was enabled by the amendment of the culture by the fungal supernatant, which, in this case, ensures a high production yield of lipopeptides. These results highlight, for the first time, that *Trichoderma harzianum* and *Bacillus velezensis* are able, in specific environmental conditions, to adapt their metabolisms in order to grow together.

## 1. Introduction

Over the last decades, coculture strategies for different microorganisms, belonging or not to the same kingdom (e.g., bacteria–bacteria or bacteria–fungus cocultures), have drawn the attention of a lot of microbiologists. This field was basically explored for its attractive results emerging on the level of the metabolism differentiation of cocultured strains [1]. Nonetheless, other interesting traits of the cocultures are also studied, such as their nutritional dependency and cross-feeding. Metabolic cross-feeding refers to the process by which one strain is capable of using a molecule that is produced by another strain as a nutrient source [2]. As for nutritional dependency, the cross-feeding is crucial for the growth of the concerned strain. For instance, the rhizosphere is known to enclose a wide variety of microorganisms interacting over nutrients. This interaction applies in the case of the mycorrhizae and the fungi-associated bacteria that grow thanks to the compounds that are produced by these eukaryotes [3]. In this regard, the uncultivability of 99% of all bacteria and archaea in laboratory conditions is partly due to the dependence of these microorganisms on nutrients or growth factors that are provided by others in their natural habitats [4,5]. In fact, according to a metabolic model that was established with 800 microbial communities, it was observed that all of the studied habitats enclose metabolically-dependent groups that swap sugars, amino acids and other metabolites [6].

In order to understand and to explore their extents, these kinds of nutritional interactions can be enforced through in vitro studies either by genetic engineering whereby a gene deletion can generate an auxotroph strain [7] or by using a culture medium that is lacking an essential nutrient for the growth of one of the cocultured species. These situations emphasize the metabolic dissimilarities which are essential for the institution of cross-feeding behavior. For example, the establishment of this type of interaction between soil bacteria belonging to the genus *Rhizobium* and actinobacteria was realized by using a defined medium containing carboxymethylcellulose as the sole carbon source that can be assimilated by the actinobacteria but not by the *Rhizobium* bacteria [8].

Bacteria and fungi that are involved in the biocontrol of plant pathogens, called biocontrol agents (BCA), are relevant candidates for coculture studies for better understanding and controlling the impact of their interactions on their biocontrol efficiency when combined. *Bacillus* and *Trichoderma* species are among the most studied BCA. They are usually found in plants’ rhizosphere and have been isolated from the roots of cucumber [5], tobacco [9] and wheat [10]. They are shown to have an antagonistic effect on plant pathogens, especially fungi such as *Fusarium*, *Sclerotinia* and many others [11,12]. Regarding *Trichoderma*, its modes of action (mycoparasitism, antibiosis and the induction of plants’ systemic resistance) and advantages (abiotic stress tolerance, high growth rate compared to fungal pathogens, etc.) as a BCA are well described by Adnan et al. [13]. On its side, *Bacillus*, especially *B. velezensis*, is well known for its high genetic capacity to produce antimicrobial molecules, especially cyclic lipopeptides [14]. Among them, surfactin, fengycin and iturin are the most commonly produced families. Some isoforms of the lipopeptides, belonging to the fengycin and iturin families, have already been described as having antimicrobial activities, which can explain the biocontrol behaviors of *Bacillus* strains [15,16].

In the past few years, only a few studies involving *Bacillus* and *Trichoderma*’s coculture have been published [17,18,19,20]. Authors have mainly focused on the influence of the coculture on the production of metabolites of interest. Thereof, they have described a boost to the production of molecules that are involved in conferring the biocontrol properties to the corresponding species. This was recently shown in terms of a *Trichoderma*–*Bacillus* interaction wherein the coculture of *Bacillus amyloliquefaciens* ACCC11060 and *Trichoderma asperellum* GDFS1009 improved the effect of the fermentation of liquor by the production of additional antimicrobial substances and amino acids [19]. The antifungal activity of this *Bacillus* strain against the fungus was not described, nor was its potential to produce lipopeptides. Furthermore, the protection of wheat alongside different *Fusarium* strains and *Botrytis cinerea* was enhanced thanks to the interaction of *T. asperellum* GDFS1009 and *B. amyloliquefaciens* 1841 [17]. The latter bacterium was suggested to produce an iturin-like compound but the effect of these potential antifungal molecules on the growth of the cocultured *Trichoderma* was not demonstrated. Moreover, a very recent case study of two other strains of *Bacillus* and *Trichoderma* brought additional evidence on the positive effect of microbial interaction on biocontrol efficacy. The coculture of these microorganisms led to the production of more antifungal molecules than in the respective monocultures [18]. Thereby, interaction between microorganisms may ensure a better biocontrol effect and growth promotion in plants. The competition between *Bacillus* and *Trichoderma* in different culture media was reported in almost all of the previous studies, without an accurate assessment of the reason behind this type of interaction. However, a more exhaustive study has highlighted the effect of the inoculation strategy on the latter species’ growth and production. The competition between *Bacillus* and *Trichoderma* was mitigated by a sequential inoculation. This led to a differential gene expression profile and, subsequently, to a higher protection capacity [21]. Even though this coculture approach impacted the expression of difficin and macrolactin by *Bacillus*, their potential activity against *Trichoderma* was not identified. In summary, all of these aforementioned studies have focused on the effect of the interaction between the different strains of *Bacillus* and *Trichoderma* on the production of secondary metabolites, using cocultures. They did not address any attention to the mechanism that is behind the observed competition between the cocultured microorganisms.

Therefore, the uniqueness of this present study is in it reporting a *T. harzianum* IHEM5437–*B. velezensis* GA1 coculture approach that is based on nutritional dependency, from which emerges a positive microbial interaction. First, the main parameter that is involved in the competition between them was determined in a coculture in a rich medium. Thus, a strategy of coculture was developed in a different nutritional condition. This condition was selected based on the nitrogen metabolism analysis of *Trichoderma* and *Bacillus*, aiming to favor the cooperation between them. The development of the microorganisms is described, as well as the effects of *Trichoderma* and its supernatant on *Bacillus*’ growth and its biosynthesis of lipopeptides through the follow-up of its lipopeptide synthetase genes’ expression. 

## 2. Materials and Methods

### 2.1. Microbial Strains

*Bacillus velezensis* GA1, formerly named *Bacillus amyloliquefaciens* GA1, was isolated from Italian strawberry fruits by the Laboratorio Vitrocoop Cesana [22], as identified by Arguelles-Arias et al. (2009) [23] and fully sequenced by Hoff et al. (2021) [24]. The strain was stored in 40% of glycerol at −80 °C. An overnight preculture in a tryptone–yeast extract medium (TY) containing 1% (*w*/*v*) tryptone, 0.5% yeast extract and 0.5% NaCl was used to inoculate the cultures. Bacteria were recovered and washed 3 times with a saline solution (0.9% (*w*/*v*) NaCl) by centrifugation and then added to the cultures so as to reach a final concentration of 2 × 10^4^ cells mL^−1^.

Six *B. velezensis* GA1 lipopeptides mutants, Δ*srfA*, Δ*fenA*, Δ*ituA*, Δ*srfA*Δ*fenA*, Δ*srfA*Δ*ituA* and Δ*ituA*Δ*fenA*, were kindly provided by the team of Prof. Marc Ongena at the University of Liege—Gembloux AgroBiotech [25]. They were stored and cultivated in the same conditions as the wild type. The recovered bacteria were washed and their concentration was adjusted to 2 × 10^4^ cells mL^−1^ in a saline solution.

*Trichoderma harzianum* IHEM5437 spores were generated on potato dextrose agar plates (PDA, Merck KGaA, Darmstadt, Germany) after 10 days of incubation at 30 °C and later kept at 4 °C. The spores were recovered with a saline solution, to which 0.01% (*v*/*v*) of Tween20 was added and they were then counted using a Bürker chamber. The spores were inoculated into the cultures so as to attain a 2 × 10^5^ spores.mL^−1^ final concentration.

### 2.2. Media and Culture Conditions

The experiments were conducted in flasks of 500 mL that were filled with 100 mL of TY medium supplemented with 0.1 mM MnCl_2_ [26] of a minimum medium (MM), the composition of which was as follows: 70 mM NaNO_3_, 7 mM KCl, 11 mM KH_2_PO_4_, 2 mM MgSO_4_ and 1% (*w*/*v*) glucose and trace elements (500× stock; 38 mM ZnSO_4_, 89 mM H_3_BO_3_, 12.5 mM MnCl_2_, 9 mM FeSO_4_, 3.55 mM CoCl_2_, 3.2 mM CuSO_4_, 3.1 mM Na_2_MoO_4_ and 87 mM EDTA) [27]. Three different cultures were performed in triplicate with the inoculation conditions that were described above: a monoculture of *Bacillus*, a monoculture of *Trichoderma* and a coculture of *Bacillus* and *Trichoderma* added simultaneously. The cultures were incubated for 1 and 6 days, respectively, in TY and MM at 30 °C and shacked at a rate of 120 rpm. The media’s pH was 6.5 and was not controlled during the culturing process. 

The *Bacillus* cultures were also conducted in supplemented MM. On one hand, the cultures were amended with 10 mM of ammonium sulfate (MM_ammonium_). On the other hand, the *Bacillus* was grown in MM in the presence of 10% (*v*/*v*) or 90% of the supernatant of *Trichoderma* or 100 g of the latter’s autoclaved mycelium. The supernatant and the mycelium came from a 6 day old culture of *Trichoderma* in MM. This supernatant was filtered through sterile CA 0.22 µm membrane filters (Sartorius Stedim Biotech GmbH, Goettingen, Germany). The mycelium was washed twice with a saline solution and autoclaved before being added to the *Bacillus* culture. The supernatant of *Trichoderma* was also fractionated through different membranes with molecular weight cut-offs of 50, 30, 10 and 3 kDa (Amicon^®^ Ultra—15, Merck Millipore Ltd., Cork, Ireland). The different fractions were later added separately to the bacterial cultures in MM at a final concentration of 10%. The addition of 90% of the *Trichoderma* supernatant to the *Bacillus* culture required the use of a 10× MM in order to maintain the same nutrient concentration in the final culture. All of the cultures were incubated for 48 h at 30 °C and shacked at a rate of 120 rpm.

The *Trichoderma* was also cultivated for 24 h in 100 mL of TY in the presence of 50 mg of surfactin, fengycin or iturin. These lipopeptides were kindly provided the team of Prof François Coutte at the University of Lille. They were produced according to the protocol that is described in Desmyttere et al. [28].

The *Trichoderma*–*Bacillus* mutants’ cultures were performed on TY agar plates. A measure of 5 µL of a 2 × 10 ^5^ spores.mL^−1^ *Trichoderma*’s solution was precultured on the plate at 30 °C. After 24 h, 2 µL of a 2 × 10 ^4^ cells mL^−1^ *Bacillus* solution (either the wild type or the mutants) was dropped from 1 cm away from the center of *Trichoderma*’s colony. The plates were incubated for 48 h at 30 °C.

### 2.3. Quantification of *Bacillus* Growth

The growth rate of the *Bacillus* bacteria was measured by following the optical density of the culture at 600 nm with a V-1200 spectrophotometer or a microplate reader (SpectraMax M2e, Molecular Devices, Sigma-Aldrich, Saint−Louis, MO, USA). In microplate reader, 96-well plates were used and incubated for 48 h at 30 °C with medium shaking. The cells were also counted by an Accuri C6 flow cytometer (BD Accuri, San Jose, CA, USA) for more accuracy. For all measurements, the coculture samples were filtered through a CA 5 µm membrane (Sartorius Stedim Biotech GmbH, Goettingen, Germany) in order to eliminate fungal mycelia and spores of any diameter up to 5 µm.

### 2.4. Scanning Electron Microscopy

Samples from the 3 culture conditions (monocultures and coculture) in MM were filtered by vacuum through a Miracloth (Millipore) filter. The filters were washed separately with gradually increasing ethanol concentrations: an overnight bath in 70% ethanol (the ethanol was changed 3 times), followed by two 30 min baths in 90% ethanol and a final bath in 100% ethanol. The samples were later dried by the use of the critical point method wherein ethanol is replaced by carbon dioxide using an Agar Scientific chamber, then sputtered with gold with a JEOL device; JFC-1100E ion sputter, fine coat. Microscopic observations were made using a SEM JEOL at a voltage of 2 kV.

### 2.5. Analytical Methods

- *Bacillus* cells’ state analysis by flow cytometry (FC):

The metabolic state of the bacteria in the MM, in both monoculture and coculture conditions, was monitored by flow cytometry using fluorescent dye. Every 24 h during the course of 6 days, samples were taken and diluted with a phosphate-buffered saline solution (PBS, 137 mM sodium chloride, 10 mM phosphate, 2.7 mM potassium chloride; pH 7.4) so as to reach a concentration that was lower than 2500 events. µL^−1^ in a 1 mL final volume. Then, 1 µL of RedoxSensor Green reagent (RSG) was added to the sample. The mix was incubated for 10 min in the dark. The measurements were conducted using an Accuri C6 flow cytometer triggering on green fluorescence (FL1 channel) which was set at a threshold FSC-H of 30,000. The flow rate was medium (35 µL.min^−1^). For each sample, 40,000 cells were assayed in order to generate statistically valid results that were expressed in terms of side scatter (SSC), forward scatter (FSC) and green fluorescence.

- *Trichoderma* supernatant composition analysis by HPLC-qTOF:

A sample of 1.5 mL of the supernatant fraction containing molecules with a molecular mass of less than 3 kDa was dried in a vacuum concentrator (Speedvac, ThermoScientific, Rochester, NY, USA). The pellet was taken up in 100 µL of water/0.1% trifluoroacetic acid and centrifuged for 10 min at 8000× *g*. Ten microliters of this solution was analyzed by HPLC (ACQUITY UPLC system, Waters Corporation) using a C18-AQ column (150 × 3 mm, 2.6 μm particles, Interchim, Montluçon, France). The elution was carried out with a flow rate of 0.5 mL.min^−1^ with a gradient that was established with solvents A and B (water + 0.1% (*v*/*v*) formic acid and acetonitrile + 0.1% (*v*/*v*) formic acid, respectively) as follows: from 1 to 30% of solvent B for 45 min, from 30 to 95% for 5 min and stabilization at 95% for 4 min. Further, the eluate was analyzed by the qTOF Synapt G2-Si™ (Waters Corporation). The molecules were, thus, ionized by electrospray at 150 °C with the capillary and cone voltages set to 3000 and 60 V, respectively, followed by the separation and detection of the *m*/*z* ratios of the ionized molecules in a coupled quadrupole analyzer to a time of flight (qTOF). The MS analysis was carried out in positive mode and in a data dependent analysis (DDA) for molecules with an *m*/*z* value between 50 Da and 2000 Da, with a scan time of 0.2 s. A maximum of 10 precursor ions were chosen for the MS/MS analysis with an intensity threshold of 10,000. The MS/MS data were collected with a CID fragmentation mode and a scan time of 0.1 s and with specified voltages ranging from 8 to 9 V and from 40 to 90 V for the lower molecular mass ions and for those with a higher molecular mass, respectively. The leucin + enkephalin ([M + H] of 556.632) was injected in the system every 2 min for 0.5 s in order to follow and to correct the measurement error during the analysis. Database searches were performed in the UniProtKB Swiss-Prot/TrEMBL database enclosing 40,820,158 proteins, via PEAKS Studio 10.6 Pro (Bioinformatics Solutions). A mass tolerance of 35 ppm and 3 missing cleavage sites were allowed. Variable methionine oxidations were also considered. The relevance of the protein and peptide identities was judged according to their scores in the research software (*p* value of 0.05 (*p* < 0.05), False Discovery Rate < 0.1%).

- Lipopeptide analysis by UPLC-MS:

Lipopeptides were assessed in the cultures’ supernatant and in the mixed biofilm in the case of the coculture. Samples were taken at the end of the cultivation and were filtrated through CA 0.22 µm membrane filters. Biofilms were recovered by centrifugation and they were then soaked in methanol for one hour for metabolite extraction. This methanol was then filtered through PES 0.22 µm membrane filters (Sartorius Stedim Biotech GmbH, Goettingen, Germany). The supernatants were further concentrated 50 times by a vacuum concentrator (Speedvac, ThermoScientific, Rochester, NY, USA).

Ultra performance liquid chromatography was conducted using an Acquity UPLC^®^ BEH C18 column 2.1 × 50 mm with a particle diameter of 1.7 µm (Waters, Milford, MA, USA) at 40 °C. The injection volume was 10 µL and the flow rate was fixed at 0.6 mL.min^−1^. The elution was performed with an initial 2.4 min gradient from 30 to 95% of solvent B (solvent A: water + 0.1% formic acid, solvent B: acetonitrile + 0.1% formic acid) followed by 2.8 min at 95% of solvent B and a final 1.8 min at 30% of solvent B. The mass of the molecules (ranging from *m*/*z* 300.00 to 2048.00) was determined by the use of an Acquity UPLC^®^ Class H SQD mass spectrometer (Waters, Milford, MA, USA) that was set in negative (ESI−) and positive modes (ES+) at a cone voltage of 60 V.

- Lipopeptide synthetase genes expression analysis by RT-qPCR:

The expression yield of the genes encoding the surfactin synthetase, fengycin synthetase and iturin synthetase was followed by real time quantitative polymerase chain reaction (RT-qPCR). RNA extractions from *Bacillus* cells in TY monoculture, MM supplemented with 90% of *Trichoderma*’s supernatant monoculture and coculture in MM were conducted using NucleoSpin^®^ RNA Midi (Macherey-Nagel, Düren, Germany). The samples were collected by a 1 min centrifugation at 10,000× *g*, following which the cells were kept in 400 µL of RNA*later* (Invitrogen, Carlsbad, CA, USA). Throughout sampling, the OD_600nm_ was measured in order to determine the growth phase in the different culture conditions. 

Luna^®^ Universal qPCR Master Mix (NEB, Beverly, MA, USA) was used to prepare the reactional mix according to the manufacturer’s instructions. The primers that were used are SrfA forward ‘attgtttacggtggctctgg’ and SrfA reverse ‘cgctgcgatagtcaaaatca’ for the surfactin synthetase gene amplification, ItuC froward ‘caagaagctctcgttacggc’ and ItuC reverse ‘gattgccggtgagatttccc’ for the iturin synthetase gene amplification and FenC forward ‘ctgaatctcttgcgccatgt’ and FenC reverse ‘tgatctgctgtgctccttca’ for the fengycin synthetase gene amplification. The expression yield was standardized according to the reference gene of gyrase for which the forward and reverse primers that were used are, respectively, ‘gagacgcactgaaatcgtga’ and ‘gccgggagacgtttaacata’. The amplification was performed using a StepOnePlusTM Real-Time PCR System thermocycler (Applied Biosystems, Foster City, CA, USA) over 5 cycles: 10 min of reverse transcription at 55 °C, 1 min of initial denaturation at 95 °C, 40 cycles of denaturation (10 s at 95 °C) and extension (30 s at 60 °C) and a final step of 1 min for melting curve generation through a gradient from 60 to 95 °C. The expression yield of the 3 synthetases gens in TY at 24 h was selected as a reference for the data comparison.

### 2.6. Metabolic Pathway Analysis

The nitrogen metabolism pathway was analyzed by Kyoto Encyclopedia of Genes and Genomes database (KEGG, https://www.genome.jp/kegg/ accessed on 10 September 2019). *B. velezensis* FZB42 and *T. reesei* QM6a were selected as the model organisms since the strains that were used in the present study are not present in the database.

### 2.7. Statistical Analysis

The statistical analyses were performed using RStudio 1.1.423 software (R language version 4.03) (Joseph Allaire, USA). A Student’s paired *t* test was adopted for comparing 2 values. The groups were considered to be significantly different at a *p*-value less than 0.05. For multiple comparisons, one-way analysis of variance (ANOVA) and Tukey’s honestly significant difference tests were performed. The groups with different letters were considered significantly different at an α-value that is less than 0.05.

## 3. Results

### 3.1. Coculture of *B. velezensis* GA1 and *T. harzianum* in Rich Medium

#### 3.1.1. Growth of Both Microorganisms in TY Medium

The monocultures of *B. velezensis* GA1 and *T. harzianum* IHEM5437 and cocultures of both were first inoculated in tryptone–yeast extract medium. In this medium, the *Bacillus* grew to reach an OD_600nm_ of 10.3 ± 0.6 after 24 h of incubation. The color of the medium turned from orange to creamy yellow.

The *Trichoderma* formed white dispersed pellets in the monoculture, reaching a dry weight of 0.79 ± 0.06 gL^−1^. The pellets were constituted of compact mycelium in a round shape. The medium remained clear orange.

In coculture, after 24 h of incubation, the bacteria were able to grow as they did in monoculture conditions (OD_600nm_ = 9.8 ± 0.4). However, the development of the *Trichoderma* could not be discerned. The medium aspect was identical to that of the monoculture of *Bacillus* and turned to creamy yellow.

#### 3.1.2. Lipopeptide Activity against *T. harzianum*

**Figure 1:**
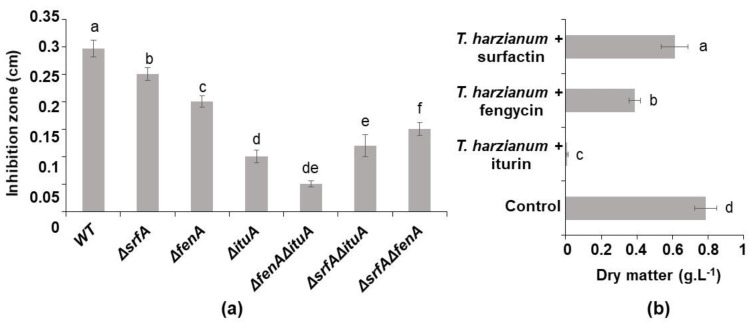
(**a**) Inhibition zone obtained in confrontation test on TY plate between *T. harzianum* and *B. velezensis* (WT: wild type) and its mutants producing one or two lipopeptides (Δ*srfA*: producing iturin and fengycin, Δ*fenA*: producing iturin and surfactin, Δ*ituA*: producing fengycin and surfactin, Δ*srfA*Δ*fenA*: producing iturin, Δ*srfA*Δ*ituA*: producing fengycin and Δ*ituA*Δ*fenA*: producing surfactin) after 48 h of incubation at 30 °C, (**b**) *T. harzianum*’s dry matter obtained after 24 h of culture in TY medium in presence of 0.5 gL^−1^ of surfactin, fengycin and iturin, respectively (all experiments were performed in triplicate). These graphs show the mean and standard deviation of three biological replicates. Different letters indicate groups of statistically different conditions (one-way ANOVA and Tukey’s HSD test (honestly significantly different); α = 0.05).

The effect of the lipopeptides that were produced by the bacteria on the growth of the *Trichoderma* was evaluated in two ways. From one side, confrontation tests between *Trichoderma* and different mutants of *B. velezensis* GA1 producing one, two or three lipopeptides at once were performed on TY plates. From another side, the direct effect of these antifungal molecules on *Trichoderma* was tested by adding purified single lipopeptides to *Trichoderma*’s culture. A clear inhibition of *T. harzianum*’s growth on the plate was noticed when the wild-type strain producing the 3 lipopeptide families was cultivated nearby (Figure 1a).

This antagonistic effect is highlighted by the inhibition zone that was found between the colonies of the respective strains. The size of the gap differed with the type of lipopeptides that were produced or not produced. When comparing the single mutants and, therefore, the effect of the lipopeptide which was not produced, the smallest inhibition zone was measured when *Bacillus* didn’t produce iturin (0.1 cm). A slightly bigger inhibition zone was obtained when fengycin was not produced and, lastly, an inhibition zone that was bigger again was produced in the absence of surfactin (0.2 and 0.25 cm, respectively). The double mutants, which produced one lipopeptide at once, displayed opposite results wherein the inhibition zone was smaller from the mutant producing only surfactin (0.05 cm) than the one producing fengycin (0.12 cm) and the one producing iturin (0.15) (Figure 1a). These results allowed the determination of the antifungal potential of each lipopeptide, despite its concentration. Iturin and fengycin showed a strong inhibitory effect. Surfactin alone didn’t display important inhibition of the development of the fungus. In order to assess their activity in accordance with their concentration, 0.50 gL^−1^ of each lipopeptide was added separately to *T. harzianum*’s culture. When 0.50 gL^−1^ of iturin was added to *Trichoderma*’s monoculture, the growth of the fungus was totally inhibited (Figure 1b). At the same concentration, fengycin had a lower inhibitory effect (50%) on the development of *T. harzianum* and surfactin showed a faint inhibition. These results are in line with those which were obtained from the confrontation tests.

#### 3.1.3. Coculture Impact on Lipopeptide Production in TY Medium

**Figure 2:**
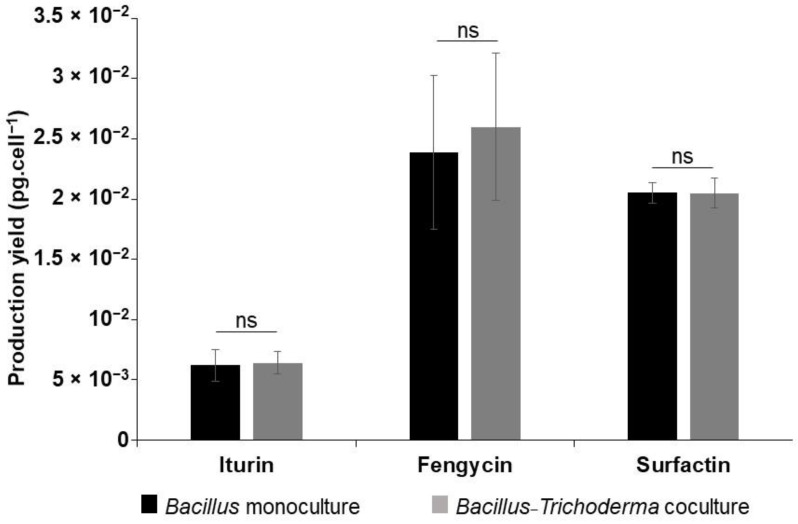
Lipopeptides’ UV spectra as generated by UPLC-MS and their respective concentrations in a 24 h old *B. velezensis* monoculture and coculture with *T. harzianum* in TY medium showing no difference in lipopeptide production profiles in these conditions. Mean values and standard deviation were calculated from three cultures (repeats). Statistical significance was calculated using Student’s paired *t* test where “ns” means no significant difference.

The production of lipopeptides by *B. velezensis* GA1, in particular the production of iturins, fengycins and surfactins, was followed by UPLC-MS. In monoculture and coculture in TY medium, the level of the production of these lipopeptides showed no significant difference (Figure 2).

For iturin, 6.2 × 10^−3^ and 6.4 × 10^−3^ pg cell^−1^ were produced, respectively, in a monoculture of *B. velezensis* and in a coculture of this bacteria with *T. harzianum*. A production yield of 2.38 × 10^−2^ and 2.59 × 10^−2^ pg cell^−1^ of fengycin was reached in these conditions, respectively. Regarding surfactin, the same production yield was obtained in both conditions (2.05 × 10^−2^ pg cell^−1^).

### 3.2. Coculture of *B. velezensis* GA1 and *T. harzianum* IHEM5437 in the Presence of a Nutritional Dependency 

In order to set up a nutritional dependency between *B. velezensis* GA1 and *T. harzianum* IHEM5437, different pathways for substrate assimilation were screened. Interesting results were shown, in particular regarding nitrogen metabolism and the ability of the microorganisms to assimilate different forms of nitrogen.

#### 3.2.1. Nitrogen Metabolism Analysis via KEGG for Culture Medium Selection

The nitrogen metabolism pathways of *T. harzianum* and *B. velezensis* were extracted from KEGG and analyzed. The assimilatory nitrate reduction pathways in these strains were compared. This pathway starts with two successive reduction reactions leading to the production of ammonium from nitrate, bypassing the production of nitrite. Thereafter, ammonium was found to be used for the production of glutamate either out of α-ketoglutarate by means of a glutamate dehydrogenase or out of glutamine and α-ketoglutarate by the glutamine synthetase/glutamate synthase cycle [29]. This glutamate was further used, essentially, for the production of biomass.

Relying on KEGG, *Trichoderma* species feature the required genes and, correspondingly, the enzymes for the assimilatory nitrate reduction. However, *B. velezensis* strains lack the nitrite reductase gene (NirA or Nit-6) and are therefore unable to use nitrate as a nitrogen source for growing. The absence of the NirA and Nit-6 genes in the *B. velezensis* GA1 genome was confirmed by a nucleotide blast (https://blast.ncbi.nlm.nih.gov/ accessed on 10 September 2019). This suggests that the presence of nitrate as a sole nitrogen source is a limiting factor for the bacterium’s growth. The availability of nitrogen in the culture medium exclusively in the form of nitrate is an interesting lead to achieve a nutritional dependency between the strains. Thereby, a defined medium (minimum medium) that was optimized for fungal culture was selected with the purpose of favoring *T. harzianum*’s growth thanks to the presence of nitrate as the sole nitrogen source.

#### 3.2.2. Growth of Both Microorganisms in Minimum Medium

In a monoculture, the *Trichoderma* grew, forming white pellets 24 h after inoculation. The pellets increased in size until day 2 of the culturing process, reaching a dry weight of 0.51 ± 0.02 gL^−1^ at day 6. 

**Figure 3:**
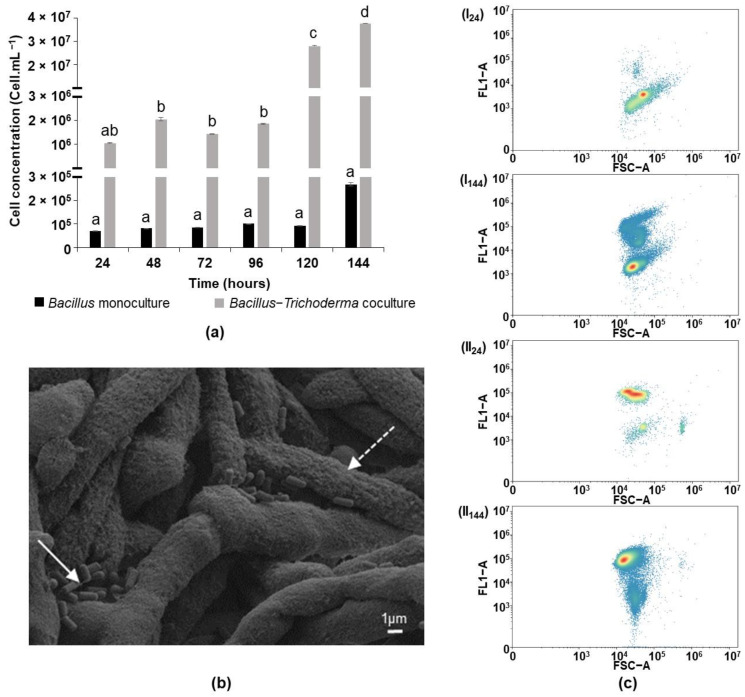
(**a**) Follow up by flow cytometry of the concentration of *B. velezensis* cells in monoculture and coculture with *T. harzianum* in MM for 6 days of culture, (**b**) SEM picture of *B. velezensis* cells (full line arrow) attached to *T. harzianum*’s mycelium (dotted line arrow) in a 6 day old coculture in MM, (**c**) Cytogram (FL1 channel for green fluorescence versus side scatter in arbitrary units) of *B. velezensis* GA1 treated with RSG after 24 and 144 h of growth in (I) monoculture in MM and (II) coculture with *T. harzianum* in MM. All the results are displayed on a FL1 dot plot on the basis of the analysis of 40,000 microbial cells by flow cytometry. The graph (**a**) shows the mean and standard deviation of three biological replicates. Different letters indicate groups of statistically different conditions (one-way ANOVA and Tukey’s HSD test (honestly significantly different); α = 0.05).

In contrast, the *Bacillus* was harder to cultivate in this medium, as predicted previously from KEGG analysis. In monoculture, the growth was slow and slight. In order to accurately follow its growth, the concentration of the *Bacillus* cells was measured every 24 h for 6 days using the flow cytometer technique. The collected data showed a 7-fold increase in the cell concentration 24 h after inoculation (Figure 3a).

After 6 days, the final cell concentration of the *Bacillus* was approximately 2.5 × 10^5^ cells mL^−1^, which corresponds to only 12.5 times more cells than were present in the initial inoculum. This concentration is not significantly different from the inoculum’s concentration.

Interestingly, in coculture with *Trichoderma* in MM, both of the strains grew. The *Trichoderma* developed pellets, as in monoculture, after 24 h; reaching a dry weight of 0.53 ± 0.05 gL^−1^ at day 6 of culturing. The bacterial cells’ concentration increased slowly in the first 96 h and reached a concentration of 1.8 × 10^6^ cells mL^−1^. Starting at day 5, a boost of *Bacillus* growth was discerned. The concentration of the *Bacillus* cells increased by more than 10 times at day 5 and an additional 1.3 times at day 6 (Figure 3a), reaching a cell concentration of 3.7 × 10^7^ cells mL^−1^.

The physical interaction between *B. velezensis* and *T. harzianum* in coculture was highlighted by scanning electron microscopy (Figure 3b). Clear attachment of the bacteria (full line arrow) on the fungal hyphae (dotted line arrow) was observed. The hyphae forming pellets were strongly entangled. Few bacteria were attached to the surface of the pellet.

The metabolic state of the *Bacillus* bacterium in monoculture and coculture was checked by FC using RSG in order to discern the impact of the presence of the fungus. The metabolic activity of the cells is reflected in terms of green fluorescence following the reduction of RSG by the enzymes that are involved in the aerobic respiration pathway [30]. The metabolic activity of cells having florescence values less than 10^4^ arbitrary units was considered to be below the detection limit, whereas active cells displayed fluorescence values higher than 10^4^. In a 24 h *Bacillus* monoculture, 95% of the cells were unable to reduce the RSG. After 6 days of incubation, 25% of the population was metabolically active. Higher activity was observed in the coculture, wherein 75 to 85% of the bacterial population was classified as being active during the 6 days of culture (Figure 3c). These results show that the presence of the fungus is essential for maintaining the metabolic activity of *Bacillus* in MM, alongside with its ability to grow.

Similar results were obtained when replacing nitrate with nitrite (data not shown).

#### 3.2.3. Microorganisms’ Growth in MM_ammonium_

Fungal and bacterial growths were possible in monoculture when the MM was amended with ammonium sulfate. The *B. velezensis* culture OD_600nm_ reached 0.95 ± 0.08. In coculture, the presence of ammonium sulfate as a nitrogen source led to the inhibition of *T. harzianum* growth by the bacterium, which developed to reach an OD_600nm_ of 0.89 ± 0.1. This confirms that nitrate and nitrite are the limiting factor for the bacterium’s growth in MM.

#### 3.2.4. *Trichoderma*’s Involvement in *Bacillus* Growth in MM

In order to investigate the reason why *T. harzianum* IHEM5437 allows *B. velezensis* GA1′s growth in MM, the fungal deactivated biomass or different concentrations of the supernatant (obtained from a 6-day old fungal monoculture) were added to the *Bacillus* inoculum. After adding *Trichoderma’s* biomass, the growth of the bacteria was slight, similar to that which was observed in the not supplemented MM. Nevertheless, adding the supernatant of *Trichoderma* to the culture medium allowed the growth of *Bacillus* in a concentration-dependent manner. The addition of 90% of the fungal supernatant to the *Bacillus* monoculture led to a higher growth of this bacterium, reaching an OD_600nm_ of 7.96 ± 0.06 in only 28 h (instead of OD_600nm_ = 0.2 ± 0.02 with a final concentration of 10% of this supernatant).

**Figure 4:**
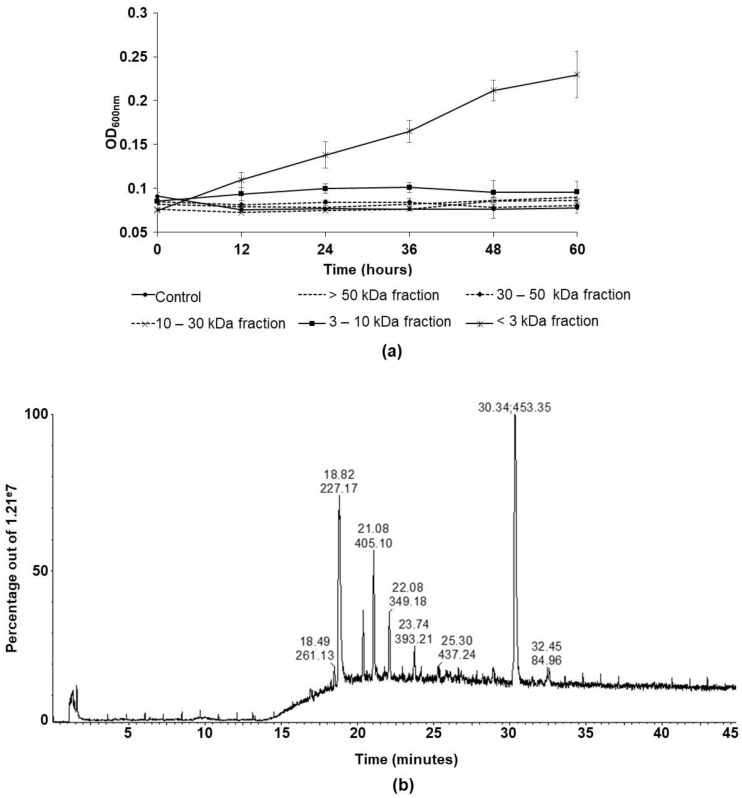
(**a**) Follow-up of *B. velezensis* growth in microplate in monocultures supplemented with different fractions of *T. harzianum*’s supernatant containing, respectively, the molecules of molecular weight higher than 50 kDa, between 30 and 50 kDa, 10 and 30 kDa, 3 and 10 kDa and lower than 3 kDa, (**b**) Total ion chromatogram obtained by RPC18-HPLC-qTOF for *T. harzianum*’s supernatant’s fraction containing molecules of molecular weight less than 3 kDa. The retention time and the value of the most intense m/z constituting each notable peak are indicated at the top of the peak.

Further analyses were conducted with the supernatant. On one hand, the fungal supernatant that was collected at day 6 was fractioned using, successively, ultrafiltration membranes with cut-offs of 50, 30, 10 and 3 kDa. This generated 5 fractions containing, respectively, molecules (if globular) of a molecular size that was higher than 50 kDa, between 30 and 50 kDa, 10 and 30 kDa, 3 and 10 kDa and finally less than 3 kDa. The fractions were added separately to the culture medium (MM) of *Bacillus* in microplates and the OD_600nm_ were obtained by a microplate reader. The growth of the *Bacillus* was noticed only in the presence of the molecules of a molecular weight that was less than 3 kDa, where the culture’s OD_600nm_ was 0.14 ± 0.06 after 48 h of culture. The growth of the bacteria when the four other fractions were added was similar to that of the control condition (without any supplementation) in which no significant growth was observed (Figure 4a).

Further analysis was conducted on the fungal supernatant’s fraction that allowed the growth of the *Bacillus* in monoculture in MM in order to potentially identify the molecules it comprises. The RPC18-HPLC-qTOF total ion chromatogram revealed, mainly, 6 intense ions with a *m*/*z* ratio equal to 227.17, 305.16, 405.10, 349.18, 393.21 and 453.35 to retention times of 18.82, 20.37, 21.08, 22.08, 23.74 and 30.34 min, respectively (Figure 4b). Unfortunately, none of these ions were identified as a peptide by MS/MS analysis. However, a lower intensity ion (*m*/*z* 531.23 and retention time 20.01 min) was identified by MS/MS analysis and spectrum confrontation in the entire UniProt database as a peptide (APDDGNMSVR) belonging to an amino-oxidase domain containing protein from *T. harzianum*.

### 3.3. Lipopeptide Production in MM

**Figure 5:**
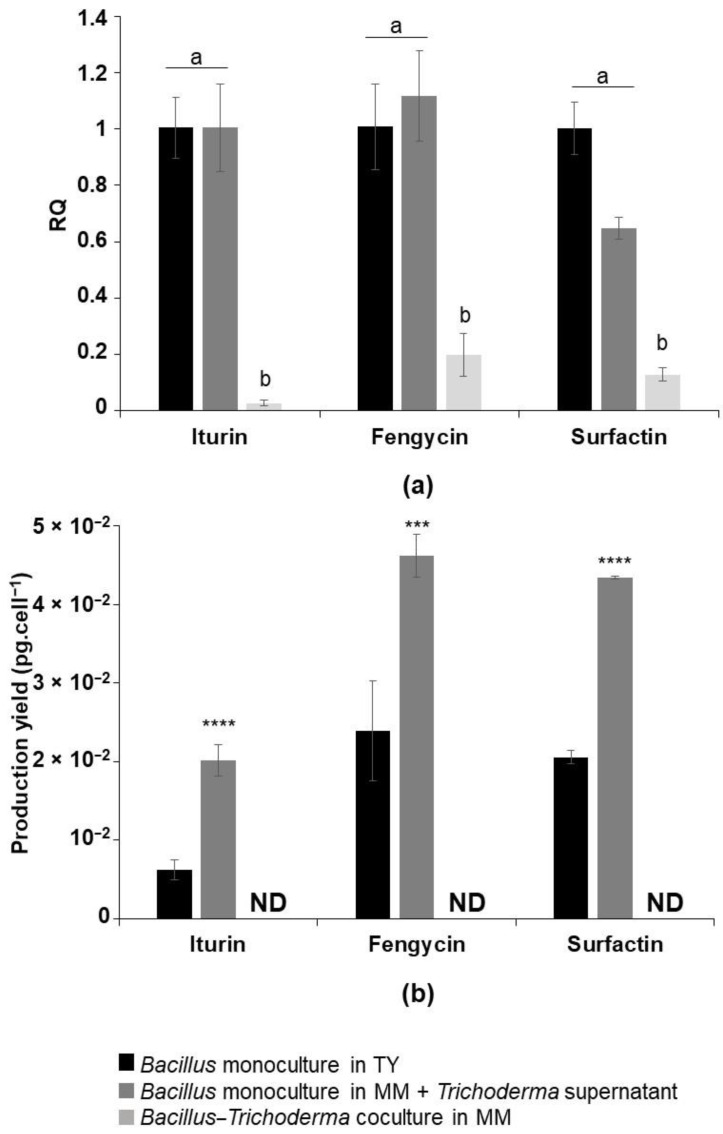
(**a**) Expression levels of iturin, fengycin and surfactin synthetases by RT-qPCR in monocultures of *B. velezensis* in TY, in MM + 90% of *Trichoderma*’s supernatant and in coculture with *T. harzianum* in MM, expressed in relative target quantity (RQ), (**b**) The respective lipopeptides concentration in the supernatant of the previous culture conditions quantified by UPLC-MS. ND = not detected. The graph (**a**) shows the mean and standard deviation of three biological replicates. Different letters indicate groups of statistically different conditions (one-way ANOVA and Tukey’s HSD test (honestly significantly different); α = 0.05). The graph (**b**) shows the mean values and standard deviation from three biological replicates. Statistical significance was calculated using Student’s paired *t* test where *** corresponds to *p* < 0.001 and **** to *p* < 0.0001.

The effect of the fungus on the production of lipopeptides by *Bacillus* was discerned by transcriptomic and metabolomic approaches. The expression of the genes of the three LPs synthetases was analyzed in coculture in MM, in monoculture in MM that was amended with 90% of *T. harzianum* supernatant and in monoculture in TY. The growth kinetics of the *Bacillus* in these three culture conditions were established for the purpose of selecting samples at the end of the exponential growth phase (data not shown). The lipopeptide concentrations were determined in the same samples by UPLC-MS. The relative target quantity (RQ) was calculated upon the RT-qPCR. The RQ for the lipopeptide synthetase genes’ expression in the *Bacillus* monoculture in TY was set at 1. The iturin, fengycin and surfactin synthetase genes were 50-, 5- and 8-fold down-regulated, respectively, in coculture, with respect to the yield that was obtained in the bacterial monoculture in TY (Figure 5a).

UPLC-MS analysis confirmed the deficit in the production of these metabolites in coculture (Figure 5b). In order to exclude any possibility of the presence of a weak undetectable concentration of lipopeptides, the supernatant of the coculture in MM was concentrated 50 times. Also, the biomass in the coculture was harvested and treated with methanol so as to allow the detachment of any potential lipopeptide that was triggered in the pellets–bacteria mixed biofilms. The UPLC analysis of the two previous samples did not reveal the detection of any lipopeptides. As a result, the production of lipopeptides is inhibited in coculture with *Trichoderma* in MM.

Furthermore, the expression of iturin, fengycin and surfactin’s synthetase genes in a bacterial monoculture that was amended with 90% of *Trichoderma*’s supernatant was investigated (the RQ for lipopeptide synthetase gene expression in the *Bacillus* monoculture in TY was set at 1). Interestingly, different expression yields of the iturin, fengycin and surfactin synthetase genes were not significantly achieved in comparison to those that were observed in the TY monoculture, while their expression was strongly down-regulated in the coculture in MM (Figure 5a). However, the production of lipopeptides by the bacteria was doubled. Indeed, in the presence of *Trichoderma*’s supernatant, 2 × 10^−2^, 4.6 × 10^−2^ and 4.3 × 10^−2^ pg cell^−1^ of iturin, fengycin and surfactin were produced, respectively, in comparison to 6.2 × 10^−3^, 2.38 × 10^−2^ and 2.05 × 10^−2^ pg cell^−1^ produced in TY and none produced in coculture in MM (Figure 5b).

## 4. Discussion

The coculture of microorganisms has been widely used for its interesting outcomes on different levels, in particular regarding the growth of uncultivable microorganisms in vitro, the expression of silent genes and the stimulation of the production of molecules of interest. Coculture refers to the cultivation of two or more microorganisms in the same medium, solid or liquid. When microorganisms belonging to different species and especially to different taxonomic domains are cocultured, the major challenge is to find culture conditions wherein they are able to grow together. As a matter of fact, it is imperative to ensure the growth of the cocultured microorganisms in order to draw interesting conclusions about the coculture’s outcome, especially on the level of the production of molecules of interest. By far, the growth and coexistence of taxonomically different microorganisms are not systematic, due to their different growth rates, competition for nutrients and the production of antimicrobial metabolites. In these cases, the faster growing or antimicrobial-producer strain suppresses the growth of the other [31].

A few studies have been carried out on the cocultures of different species of *Bacillus* and *Trichoderma*. These studies describe the technical aspect of the coculture, its effect on certain traits of the strains by a transcriptional approach, the production of molecules of interest and their biocontrol activity. Competition between the two strains has also been observed, even when a sequential inoculation strategy was adopted in order to guarantee the growth of both of the microorganisms [21]. It was also proven that the interaction between *T. virens* GI006 and *B. velezensis* BS006 on solid media varies depending on the composition of the culture medium [20]. Even though the effect of the supernatant of each microorganism on different growth parameters of the other was studied, the compatibility between these strains in a liquid coculture was not shown.

In this work, we demonstrated that the compatibility between *B. velezensis* and *T. harzianum* is highly dependent on the nutritional conditions of the culture medium. In fact, the growth of *Trichoderma* is inhibited by *B. velezensis* in coculture in a medium wherein all of the required nutrients for the development of both of the strains are available. In this condition, these microorganisms are not compatible. We assigned this inhibition to the antifungal lipopeptides that are produced by *B. velezensis* GA1, notably those belonging to the family of iturins and, to a lesser extent, to those from the family of fengycins. The greatest inhibition of *T. harzianum* was recorded with a *B. velezensis* GA1 mutant producing both fengycin and iturin simultaneously, followed by the mutants producing only iturins and fengycins, respectively. The inhibition by these double mutants remained lower compared to that which was caused by the single mutant strains. This indicates the additional activity of fengycin and iturin. Similarly, these two lipopeptides, which are produced by *B. velezensis* Y6 and F7, were also selected as the metabolites with the most important antifungal activity against *R. solanacearum* and *F. oxysporum* [32]. Fengycins and iturins are known in particular for their antifungal activity related to their amphiphilic structure. This allows them to interact with sterols and phospholipids in the fungal membrane. This action damages the cell membrane by creating pores and makes the cells more permeable and sensitive to other antifungal molecules [33,34]. To conclude, the coculture of *T. harzianum* and *B. velezensis* strains in a rich medium led to a competitive interspecific interaction that did not allow their coexistence, nor affected the lipopeptides’ production.

Hereby, we implemented a different approach in order to fill the gap that was remaining in the *Trichoderma*–*Bacillus* research field. The compatibility between *T. harzianum* and *B. velezensis* was studied using a coculture strategy that was based on the creation of a nutritional dependency. The term “nutritional dependency” means that only one species is able to consume an essential substrate. Consequently, the metabolites that are produced by this species will be used by the second species in order to ensure its growth. The latter is then unable to develop in monoculture under the same conditions [35]. In fact, the acquisition of nutrients is the major reason for the establishment of competition between microorganisms. Especially in growing media wherein resources are limited, microorganisms struggle to acquire their nutritional needs and the one that is more efficient will succeed in invading the other [36]. In coculture, under conditions where one of the two competitive microorganisms does not have a given element that is essential to its growth and the other microorganism must provide it, the competitive relationship may evolve towards commensalism [37]. Mutualism can also be observed if the nutritional dependency is bidirectional [38]. Thereby, the nutrient interdependency can help to reshape the type of microbial interaction [37]. In other words, two incompatible microorganisms can coexist without competition if the growth of one is dependent on the other for nutritional reasons.

For the purpose of creating a nutritional dependency between *B. velezensis* and *T. harzianum*, their different metabolic pathways were analyzed by KEGG. A complementary metabolic relationship between them was noticed at the nitrogen metabolism level. Indeed, *B. velezensis* seems to be unable to use nitrate or nitrite as its sole source of nitrogen due to the absence of the nitrite reductase encoding gene, unlike *Trichoderma*. This enzyme is essential for the conversion of nitrate or nitrite to ammonium for the biosynthesis of nucleic acids and amino acids [39]. Therefore, the absence of the biosynthetic function enabling *Bacillus* to exploit nitrate as nitrogen source, thus preventing its growth in monoculture, would be compensated by its coculture with *Trichoderma*.

Hence, a defined medium (MM) comprising nitrate as the sole nitrogen source was selected in order to better tailor the interaction between the microorganisms. The use of this key substrate created a nutritional dependency between the cocultured microorganisms. This was confirmed by analyzing the growth of *B. velezensis* GA1 and *T. harzianum* IHEM5437 in monocultures and coculture in MM. Compared to the rich medium, the fungus grew at lower levels in this medium. This decrease is explained by the form of the available nitrogen source. Although *Trichoderma* can assimilate nitrate, this form is not optimal for its growth [40]. As for *B. velezensis*, the maximal level of biomass that was reached in monoculture in MM was 10^6^ times lower than the level that it reached in the TY medium. Its growth was not significant compared to that of the bacterial inoculum. The incapacity of *Bacillus* to grow in this medium was compensated by adding another nitrogen source, ammonium, that seems to be easily assimilated by the bacterium. Also, *B. velezensis* GA1 successively developed in the MM in the presence of *T. harzianum*. In this condition, the bacterial cells remained metabolically active which reflects their viability and their ability to produce metabolites. The *Bacillus* cells also showed an ability to attach to the fungal mycelia. This behavior is reported in several studies and seems to depend on several factors such as the viability of the fungus, its stage of growth and the region to be colonized. For instance, the attachment of *B. cereus* VA1 was favored on degraded hyphae whereas *B. subtilis* grows on the mycelial areas of *Aspergillus niger* which are producing more protein [26,41]. Thus, the presence of *Trichoderma* positively influenced the bacterium in the MM in coculture, compared to in a monoculture. To summarize, this work reports for the first time a *Bacillus–Trichoderma* coculture strategy in a liquid medium wherein the microorganisms are compatible in terms of their growth.

This strategy implements a delay between the development of the microorganisms, which is essential for their co-development. Once the *Trichoderma* had grown, it produced metabolites that allowed, subsequently, for the growth of the *Bacillus*. Furthermore, the development of this bacterium is dependent on the quantity of the supernatant that is added to its monoculture and, subsequently, to the quantity of the molecules of interest that are provided. Interestingly, it was observed that among these molecules of a molecular weight less than 3 kDa one was identified as a peptide which resulted from the hydrolysis of proteins containing amino-oxidase domains. These are believed to be nitrogen sources for the bacterium. In fact, *Trichoderma* strains are widely explored for their high potential to produce amino acids and proteins [42]. These molecules, considered as organic forms of nitrogen, may serve as nutriments for *Bacillus* allowing its growth in the presence of nitrate as the sole nitrogen source.

Nevertheless, the coculture of *B. velezensis* GA1 with *T. harzianum* IHEM5437 in MM engendered an inhibition of the production of the bacterial lipopeptides through the repression of the expression of the respective synthetase genes. Examples in the literature have shown different production profiles of lipopeptides by *Bacillus* strains in the presence of other microorganisms with which they have or have not established direct contact. The production was increased in the presence of the molecules that were produced by the pathogenic fungus *Rhizomucor* and in coculture with *Pythium* and *Fusarium* [43]. On the other hand, the reduction in the synthesis of molecules with antibiotic activity in a coculture is also common and promotes the coexistence of the cocultured microorganisms [44]. This reduction is generally based on the regulation of the expression of the corresponding genes by the exchanged molecules. For instance, within the framework of the interaction between *B. subtilis* and *Aspergillus niger*, the expression of the surfactin synthetase operon, as well as the production of this metabolite, were strongly reduced in coculture [26]. An analogous regulation loop was discerned in *T. atroviride* and *B. amyloliquefaciens*’ interaction wherein the fungus *Vel1* gene was overexpressed in the presence of *Bacillus*, leading to the down-regulation of the expression of the polyketides synthases genes (difficidin and macrolactin) in the bacterium [45]. Likewise, it is possible that a similar regulation loop is set in the interaction between *T. harzianum* IHEM5437 and *B. velezensis* GA1. Additionally, this regulation requires the simultaneous presence of both species because adding the fungal supernatant induced the production of lipopeptides in a *Bacillus* monoculture in MM. It can be suggested that the inhibition of the production of *B. velezensis*’ lipopeptides by *T. harzianum* is due to the signals that are exchanged between the microorganisms. These exchanges comprise, from one side, the perception of *Bacillus*’ signals by *Trichoderma* and, from another side, the production of signals by *Trichoderma* regulating the expression of lipopeptide synthetase genes in *Bacillus*. Such an interpretation was also observed in the interaction between *B. velezensis* S499 and the pathogen *R. variabilis*, wherein the perception of some pathogens’ molecules by *Bacillus* induced the production of fengycins [43]. 

## 5. Conclusions

A new approach was developed in order to successfully coculture a fungus of the genus *Trichoderma* and a bacterium from the genus of *Bacillus* that was not able to assimilate nitrate. This approach was based on setting up a nutritional dependency between the microorganisms which helped to shift their behavior from competitive to commensal. *T. harzianum* is able to produce molecules of interest that were clearly acting as nitrogen sources. These were essential for the growth of *B. velezensis* in this condition. The intricacy of this interspecific interaction is underlined by the repression of the genes that encode lipopeptide synthetases in *B. velezensis* when both of the microorganisms are cultured together. However, they were not detected when *B. velezensis* was in the presence of the fungal supernatant; in other words, in absence of the previous contact between them. The latter finding can be exploited in the biocontrol field wherein the use of lipopeptides to control phytopathogens is widening. 

## Data Availability

Data related to this paper may be requested from the authors.
